# Haemodynamic changes during alveolar recruitment manoeuvre in patients with diastolic dysfunction

**DOI:** 10.1186/cc12046

**Published:** 2013-03-19

**Authors:** A Rubino, E Marini, B Ferro, M Collareta, F Forfori, F Guarracino, F Giunta

**Affiliations:** 1 Azienda Ospedaliero Universitaria Pisana, Pisa, Italy

## Introduction

To investigate the haemodynamic effects of an alveolar recruitment manoeuvre in intubated patients with diastolic dysfunction compared with patients with normal diastolic function in a mixed ICU.

## Methods

Between October 2011 and February 2012, 16 mechanically ventilated patients admitted to the ICU with normal systolic function and without inotropic support were enrolled. A transthoracic echo-cardiography was performed to assess diastolic function: E/A, isovolumic relaxation time (IVRT), deceleration time (DT), E/e' were assessed. Eight patients (five with poor relaxation pattern, three with decreased left ventricle compliance) were identified in diastolic dysfunction group (Group 1) and eight were included in the normal diastolic function group (Group 2). A recruitment manoeuvre was performed by applying a PEEP of 40 cmH_2_O for 30 seconds. Haemodynamic parameters of cardiac output (CO), stroke volume (SV), heart rate (HR), pulse pressure variation (PPV), and cardiac cycle efficiency (CCE) were continuously recorded during the manoeuvre using the MostCare pulse contour method.

## Results

No significant haemodynamic changes happened in the first part of the recruitment (t0 to t15 seconds). A significant decrease in CO (*P <*0.05), SV (*P <*0.01), systolic pressure (*P <*0.04) and PPV (*P *< 0.04) occurred in Group 1 during the remaining part of the manoeuvre. No significant decrease in the same haemodynamic parameters were noted when only E/A was used to discriminate the diastolic dysfunction. See Table 1.

**Table 1:** CO difference associated with diastolic dysfunction parameters

	E/A <1	E/A >1	*P *value
CO 15 to 30	3.9	4.8	0.20
	E/e' <1	E/e' >1	
CO 15 to 30	3.92	5.6	0.02

## Conclusion

The recruitment manoeuvre can compromise left diastolic filling by increasing the transpulmonary pressure. In patients with diastolic dysfunction a significant decrease in SV can be observed during the recruitment manoeuvre with no compensatory mechanisms evoked determining a significant decrease in CO. The E/A ratio is not able to discriminate between the two groups, confirming the power of tissue Doppler imaging to recognize the correct diastolic pattern.
